# A Rare Case of Live Birth Through In Vitro Fertilization in a 46-Year-Old Woman Using Her Autologous Oocytes: Case Report and Literature Review

**DOI:** 10.1155/carm/5531403

**Published:** 2025-07-05

**Authors:** Hossam Elzeiny

**Affiliations:** ^1^Reproductive Services Unit, Royal Women's Hospital, Melbourne, Victoria, Australia; ^2^City Fertility Centre, East Melbourne, Melbourne, Victoria, Australia

**Keywords:** advanced maternal age, ICSI, live birth, oldest autologous IVF, oldest mother over age 45 in IVF

## Abstract

Female fertility decline with age presents a challenge to successful IVF outcomes. The rising trend of delayed family planning due to societal changes has led to increased demand for reproductive assistance among older women. Despite clinical and scientific advances in Assisted Reproductive Technology, age remains a barrier to successful outcomes mainly due to declining oocyte quality and quantity, leading to decreased fecundity rates and increased miscarriage risks. This report highlights an exceptional case of a women aged 46 achieving live birth through IVF using her own oocytes. Notably, awareness among women about the profound effect of age on fertility remains insufficient. Educating women about these age-related reproductive challenges is crucial. Oocyte cryopreservation emerges as a potential strategy, while egg donation stands as a pragmatic alternative. However, the probability of live birth among women at the extremes of reproductive age using their own oocytes is low, at 0.3%. It is important to approach this case report with caution to avoid unrealistic expectations among women aged 45 or older seeking IVF services.

## 1. Introduction

In the field of human reproduction, the limited span of female fertility, defined by age, has not paralleled the remarkable increase in longevity witnessed by women in recent years. The onset of childbearing has significantly shifted since the contraceptive revolution of the 1960s, leading to a prevailing trend of starting families at later stages of life [1].

Advanced maternal age (AMA), generally regarded as commencing at 35 years old, extends to very AMA (VAMA), typically defined as ≥ 40 years, and extremely AMA (EAMA) delineates women aged ≥ 45 years [2]. This age categorization marks a significant biological decline in both the quantity and quality of oocytes. This decline not only results in decreased fecundity rates but also increases the risk of miscarriage due to chromosomal abnormalities [3]. By the mid-forties, the reproductive window closes, rendering any subsequent fertility treatment futile. Despite remarkable advancements in in vitro fertilization (IVF) technology, age remains a substantial challenge for successful outcomes. Molecular mechanisms underlying oocyte aging include, but are not limited to, telomere shortening, errors in meiotic recombination, mitochondrial dysfunction, disorders of protein homeostasis, oxidative stress [4], noncoding RNA, autophagy and DNA damage, and the emerging concept of the epigenetic clock [5].

While the management of these mechanisms remains under ongoing research, current reality necessitates a reliance on egg donation or adoption as the only viable solutions for women of AMA. IVF fails to compensate for the detrimental effects associated with AMA, and predictive factors for success in this context remain elusive. Alarming studies have highlighted the lack of awareness among most women regarding the heightened risk of infertility due to delayed childbearing [6]. Additionally, many women hold misconceptions that IVF can invariably address fertility decline linked to advancing age [7], while some believe in an age limit beyond which conception is deemed unattainable [8].

Studies have demonstrated an inverse U-curve pattern of natural fertility in both young women and those approaching AMA [9]. In an attempt to address the complexities surrounding IVF treatment and advanced age, various IVF centres worldwide have developed their own guidelines to determine the cut-off age for offering IVF using a woman's own oocytes. In our centre, the cut-off age is set at the 46th birthday. In this report, we describe a compelling case where a woman approached our centre a few days prior to her 46th birthday, seeking to initiate her final IVF cycle using her own oocytes. To date, only six published cases have reported successful live births (LB) at the age of 46 [10–15].

## 2. Case Presentation

**Figure 1:**
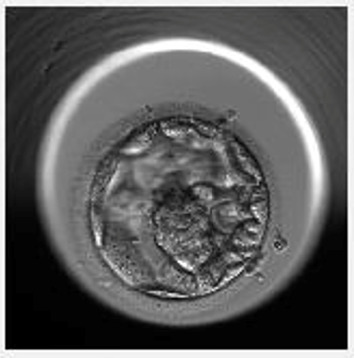
Embryoscopic view of 4AA blastocyst at 112 h postinsemination.

In 2022, a 45-year-old woman presented to our centre due to secondary infertility concerns. Her obstetric history indicated gravida1, para1, abortion 0. Her first successful conception occurred through IVF at the age of 43, motivating her to pursue IVF treatment once more to expand her family. She reported menarche at 15 years old, maintained a regular 28-day cycle. At the time of presentation, her BMI was 22.91 kg/m^2^, as shown in Table 1 and there were no relevant medical, surgical, or family histories of note. Evaluation of her ovarian reserve revealed an AMH of 3.5 pmol/L and a total Antral Follicle Count (AFC) of 5. The patient underwent preconception counselling with a high-risk obstetrician and comprehensive medical evaluation to assess her suitability for pregnancy. In April 2022, a short flare protocol was initiated from the second day of the menstrual cycle by nasal administration of 200 mcg twice daily of GnRH agonist (Nafarelin-Synarel; Pfizer, Ballerup, Denmark). Followed by rFSH plus rLH (Pergoveris, Serono, Darmstat, Germany) administration at a dosage of 450 IU per day, began on the third day of the cycle until the day of HCG administration. Follicle development was monitored by transvaginal ultrasound. On cycle day 8, transvaginal ultrasound revealed six developing follicles with mean diameters of 18, 16, 16, 13, 10, and 10 mm, accompanied by an endometrial thickness of 10 mm exhibiting a trilaminar appearance. Serum hormone analysis demonstrated an oestradiol concentration of 4820 pmol/L and a progesterone level of 2.8 nmol/L. Ovulation was subsequently triggered on cycle day 9 with 250 μg of recombinant hCG (Ovidrel; Merck, Macquarie Park, Australia) to induce final oocyte maturation. Oocyte retrieval was planned 37 h after trigger using a 17-gauge/35 cm single-lumen needle (Cook Medical, Eight Mile Plains Queensland, Australia) through transvaginal follicle aspiration under ultrasound guidance. Six oocytes were retrieved of those four were mature. A single motile sperm was selected and injected into each of the four metaphase II oocytes. Three oocytes were fertilized normally, while one embryo arrested. The remaining two embryos developed into blastocysts by day 5 postfertilization. The first blastocyst, graded as 4AA according to the Gardner scoring system [16], was suitable for fresh embryo transfer (Figure 1). The second blastocyst, graded as 4AB, was cryopreserved on day 5 postfertilization for future use. The embryo transfer procedure was performed under abdominal ultrasound guidance using the Cook Sydney IVF Embryo Transfer Set (Cook Medical, USA) catheter, and the blastocyst was transferred in approximately 20 μL of Embryo Glue (Vitrolife). Luteal phase support was initiated 2 days after oocyte retrieval using progesterone gel (Crinone 90 mg twice daily, Merck, Macquarie Park, Australia).

The patient's β-hCG level was positive (252 mIU/mL) nine days after embryo transfer, confirming biochemical pregnancy. Patient age on positive BHCG day was 46 yo and 10 days. At 6 weeks and 4 days of gestation, a single intrauterine gestational sac with fetal heartbeat was documented via transvaginal ultrasound, confirming a viable pregnancy. The pregnancy antenatal course was uneventful and patient successfully delivered a live male offspring weighing 3220 g via caesarean section (due to failure to progress) at 39 weeks and 4 days of gestation in January 2023. The newborn showed no apparent abnormalities.

## 3. Discussion

This case provides unique insight into successful autologous IVF in a woman, aged 46 contributing novel clinical data to a scant body of evidence on such rare outcomes. Only 0.2% of all deliveries result from spontaneous pregnancies in women aged 45 years and older [17]. To the best of our knowledge, only six cases have been documented to date [10–15], highlighting the rarity and significance of these occurrences. Pregnancy outcomes are summarized in Table 2. This detail is clinically significant as prior cases, often involved, cleavage stage embryo transfers or lacked specification of embryo stage. The successful use of blastocyst stage embryo suggests that even in severely diminished ovarian reserve embryo development to blastocysts stage is possible and it may offer a feasible route for embryo selection, and improved implantation in women of extremely advanced reproductive age. This observation prompts further investigation into the biological, and possibly genetic factors enabling such successful conception at an AMA.

Importantly, our case is the first reported instance of a LB achieved via blastocyst stage embryo transfer in a woman aged 46 using autologous oocytes. This underscores the importance of long-term observational data and follow-up registers to assist both maternal and offspring outcomes.

It is crucial to acknowledge the physiological changes that occur with age, particularly in women. As women age, their ovarian reserve decreases, resulting in diminished fertility and an increased risk of chromosomal abnormalities in the offspring. This decline in fertility is primarily attributed to the decreased quality and quantity of oocytes available for fertilization [9]; recently, a role of endometrial aging as a result of altered gene expression has been proposed [18]. Consequently, achieving a successful pregnancy and LB becomes increasingly challenging as women approach and surpass the menopausal age [19].

The largest single-centre report [14], documented a LB rate of 1/268 (0.37%) in all initiated natural IVF cycles, while a report by Gunnala [15] shows a LB rate per initiated autologous IVF cycle of 1/221 (0.45%) for women aged 46 years, Notably, in the specified age group, there is currently no widely endorsed and efficacious stimulation protocol [20]. In fact, in this age bracket, IVF yields result comparable to those of natural conception [21].

In our report, AMH at this age group has no correlation with oocyte quality [22], our case underwent conventional IVF protocols, encompassing flare protocol, no adjuvants were added. Given the expected, low ovarian response and lower blastulation rate and higher probability of aneuploidy, preimplantation genetic testing for aneuploidy (PGT-A) was offered but declined by patient. However, after careful counselling prenatal genetic testing was advised and this approach agreed with previously reported pregnancies in the same age group [14]. It is worth noting that our case was the first to achieve LB using blastocyst stage embryo transfer.

Additionally, it is essential to consider the potential risks associated with late-age pregnancies for both the mothers and their offspring. AMA is associated with an increased risk of obstetric complications, such as gestational diabetes, hypertension, placental abnormalities, and an elevated risk of caesarean section [23]. Furthermore, the offspring of older mothers may be more susceptible to genetic disorders, such as Down syndrome and other chromosomal abnormalities, as well as an increased risk of neurodevelopmental disorders [3].

Data from the most recent annual report of the Australia and New Zealand Assisted Reproduction Database [24] for outcomes of autologous fresh cycles by female age in 2021 show that 1547 IVF cycles initiated for women > 45 yo resulted in 1.4% LB per initiated cycle using autologous fresh cycle but there was no stratification for age of 46. On the other side of the globe, the most recent national clinical summary report published by the Society for Assisted Reproductive Technology [25]. An affiliated body of the American Society for Reproductive Medicine (ASRM) indicates a 4% LB rate from initiated autologous 9805 cycles for women > 42 years, but no data are provided for specific years in this older age range [26]. Additionally, data from the United States Department of Health and Human Services showing the birth rate for women aged 45–49 (includes births to women aged 50 and over) was 0.9 births in 2021; again, there were no stratification for age and it was not clear whether these were natural or IVF pregnancies. Similarly, The European IVF Monitoring Consortium (EIM), for The European Society for Human Reproduction and Embryology (ESHRE), reporting on the year 2018 [27], provided LB data for an age up to 45 years but nil documented thereafter and there was no distinction between using frozen embryos created from oocytes retrieved at a younger age.

The precedent for the most AMA resulting in a LB through IVF is held by a 49-year-old woman who gave birth at the age of 50 from West Bengal, India [28]. However, scepticism is warranted due to potential limitations in accurate age documentation. In a recent publication [29], documented a LB in a 48-year-old woman through IVF using autologous oocyte.

In light of these potential risks, it is crucial to evaluate the thoroughness of the preconception counselling, prenatal care, and screening protocols that were implemented in these cases. It is important to determine whether appropriate assessments were conducted to evaluate the overall health of the mothers and to identify any potential risk factors or complications that may arise during pregnancy. Understanding the management strategies employed in these cases can provide valuable insights into optimizing the care for women pursuing late-age pregnancies.

Moreover, ethical considerations arise regarding the welfare of both the mothers and the children. Late-age pregnancies raise questions about the long-term well-being of the mothers, as they may face increased physical and emotional challenges associated with caring for a child at an advanced age. Additionally, the potential impact on the children's well-being, including their social development, parental support, and longevity of maternal care, should be carefully examined [30].

The limited number of reported cases highlights the need for comprehensive long-term follow-up studies to assess the physical and psychological outcomes for both the mothers and the children. Such studies can provide a better understanding of the long-term implications and potential health risks associated with pregnancies at an advanced age, informing future medical decisions and counselling for women considering late-age pregnancies [31].

Oocyte cryopreservation at a younger age has emerged as a firmly established strategy for mitigating the deleterious impact of aging [32].

In cases where egg cryopreservation is not pursued or unsuccessful in achieving a LB, oocyte donation stands as the primary and pragmatic treatment option [33].

## 4. Conclusion

This case presented here illustrates the rare but remarkable possibility of achieving a successful LB at the age of 46 using autologous oocytes and standard IVF techniques. While such outcomes are exceptional, they highlight that LB is not impossible even in markedly diminished ovarian reserve and AMA. The likelihood of a LB for women at the far ends of their reproductive age using their own oocytes stands at a mere 0.3%. Nevertheless, this case also serves to reinforce the importance of early fertility counselling, including timely consideration of elective oocyte cryopreservation or the use of donor oocytes when appropriate. In an era of delayed childbearing, this report contributes valuable insight into the limits and possibilities of assisted reproductive technology.

## Figures and Tables

**Table 1:** Demographics and cycle characteristics summary.

Patient demographics and cycle characteristics	Fresh IVF cycle
Age at OPU	46 yo-5 days
Type of infertility	Secondary
Duration of infertility	N/A
No. of previous IVF cycles	1
BMI	22.91
AFC	5
Type of stimulation protocol	Flare Pergoveris 450 IU
No. of eggs retrieved	6
Type of fertilization	ICSI
No. of mature eggs	4
Fertilisation rate (%)	3/4 (75%)
No. and day of embryo transfer	Single D5
Surplus vitrified embryos	One blastocyst
Initial BHCG 9 days after ET	252 IU
Duration of pregnancy	39 w + 4 d
Mode of delivery	C/S
Birthweight	3220 gm
Sex of the baby	Male

**Table 2:** IVF outcome of women conceived at the age of 46 using autologous oocytes.

Study	Stim protocol	# of oocytes	Day of ET	# ET	Live birth
Prato et al. [12]	Clomid 150	3	2	1	Male
Check et al. [13]	N/A	N/A	N/A	N/A	Female
Trolice [14]	FSH 300 and Letrozole	7	3	4	Male 31 w
Gunnala et al. [15]	Not mentioned	N/A	3	> 2	1/221 N/A
Fujishiro et al. [16]	Natural IVF	2	2	Single	Female
Yoshida et al. [17]	Flare HMG	3	4	3	Male 40 w 2 d

## Data Availability

The data that support the findings of this study are available from the corresponding author upon reasonable request.

## References

[1] Llarena N., Hine C. (2021). Reproductive Longevity and Aging: Geroscience Approaches to Maintain Long-Term Ovarian Fitness. *The Journals of Gerontology: Series A*.

[2] Seshadri S., Morris G., Serhal P., Saab W. (2021). Assisted Conception in Women of Advanced Maternal Age. *Best Practice & Research Clinical Obstetrics & Gynaecology*.

[3] Franasiak J. M., Forman E. J., Hong K. H. (2014). The Nature of Aneuploidy With Increasing Age of the Female Partner: A Review of 15,169 Consecutive Trophectoderm Biopsies Evaluated With Comprehensive Chromosomal Screening. *Fertility and Sterility*.

[4] Wang X., Wang L., Xiang W. (2023). Mechanisms of Ovarian Aging in Women: A Review. *Journal of Ovarian Research*.

[5] Li Piani L., Vigano P., Somigliana E. (2023). Epigenetic Clocks and Female Fertility Timeline: A New Approach to an Old Issue?. *Frontiers in Cell and Developmental Biology*.

[6] Mac Dougall K., Beyene Y., Nachtigall R. D. (2013). Age Shock: Misperceptions of the Impact of Age on Fertility Before and After IVF in Women Who Conceived After Age 40. *Human Reproduction*.

[7] Fauser B. C. J. M., Boivin J., Barri P. N., Tarlatzis B. C., Schmidt L., Levy-Toledano R. (2019). Beliefs, Attitudes and Funding of Assisted Reproductive Technology: Public Perception of Over 6,000 Respondents From 6 European Countries. *PLoS One*.

[8] Billari F. C., Goisis A., Liefbroer A. C. (2011). Social Age Deadlines for the Childbearing of Women and Men. *Human Reproduction*.

[9] Gruhn J. R., Zielinska A. P., Shukla V. (2019). Chromosome Errors in Human Eggs Shape Natural Fertility Over Reproductive Life Span. *Science*.

[10] Gardner D. K., Lane M., Stevens J., Schlenker T., Schoolcraft W. B. (2000). Blastocyst Score Affects Implantation and Pregnancy Outcome: Towards a Single Blastocyst Transfer. *Fertility and Sterility*.

[11] Laufer N., Simon A., Samueloff A., Yaffe H., Milwidsky A., Gielchinsky Y. (2004). Successful Spontaneous Pregnancies in Women Older Than 45 Years. *Fertility and Sterility*.

[12] Prato L. D., Borini A., Cattoli M., Preti M. S., Serrao L., Flamigni C. (2005). Live Birth After IVF in a 46 Year-Old Woman. *Reproductive BioMedicine Online*.

[13] Check J. H., Chern R., Amui J. (2011). Successful Pregnancy Following In Vitro Fertilization Embryo Transfer in a 46 Year-Old Woman With Diminished Oocyte Reserve as Evidenced by a High Day 3 Serum Estradiol. *Clinical & Experimental Obstetrics & Gynecology*.

[14] Trolice M. P. (2014). Live Birth from a 46 Year-Old Using Fresh Autologous Oocytes Through In Vitro Fertilization. *Fertility and Sterility*.

[15] Gunnala V., Irani M., Melnick A., Rosenwaks Z., Spandorfer S. (2018). One Thousand Seventy-Eight Autologous IVF Cycles in Women 45 Years and Older: The Largest Single-Center Cohort to Date. *Journal of Assisted Reproduction and Genetics*.

[16] Fujishiro E., Yoneyama K., Kakinuma T., Kagawa A., Tanaka R., Kaijima H. (2021). Retrospective Outcome in Women Aged 45 Years and Older Undergoing Natural Cycle IVF Treatment. *Reproductive BioMedicine Online*.

[17] Yoshida H., Oomiya Y., Sato T., Aono N., Araki Y. (2004). A Successful Pregnancy and Delivery Outcome for a 46 Year-Old Woman Following In Vitro Fertilization. *Reproductive Medicine and Biology*.

[18] Vitagliano A., Paffoni A., Viganò P. (2023). Does Maternal Age Affect Assisted Reproduction Technology Success Rates After Euploid Embryo Transfer? A Systematic Review and Meta-Analysis. *Fertility and Sterility*.

[19] Eijkemans M. J., van Poppel F., Habbema D. F., Smith K. R., Leridon H., Velde E. R. (2014). Too Old to Have Children? Lessons From Natural Fertility Populations. *Human Reproduction*.

[20] Pal A., Mani T., Chinta P., Karthikeyan M., Kunjummen A. T., Kamath M. S. (2023). Effectiveness of GnRH Agonist Short Protocol Versus GnRH Antagonist Protocol in POSEIDON Groups 3 and 4: A Retrospective Cohort Study. *Reproductive Sciences*.

[21] Spandorfer S. D., Bendikson K., Dragisic K., Schattman G., Davis O. K., Rosenwaks Z. (2007). Outcome of In Vitro Fertilization in Women 45 Years and Older Who Use Autologous Oocytes. *Fertility and Sterility*.

[22] Dai X., Wang Y., Yang H. (2020). AMH Has No Role in Predicting Oocyte Quality in Women With Advanced Age Undergoing IVF/ICSI Cycles. *Scientific Reports*.

[23] Sugai S., Nishijima K., Haino K., Yoshihara K. (2023). Pregnancy Outcomes at Maternal Age Over 45 Years: A Systematic Review and Meta-Analysis. *American Journal of Obstetrics & Gynecology MFM*.

[24] Newman J. E., Paul R. C., Chambers G. M. (2021). *Assisted Reproductive Technology in Australia and New Zealand*.

[25] Sart (2020). National Summary Report for the Year. https://www.sartcorsonline.com/rptCSR_PublicMultYear.aspx.

[26] Osterman M. J. K., Hamilton B. E., Martin J. A., Driscoll A. K., Valenzuela C. P. (2023). Births: Final Data for 2021. *National Vital Statistics Reports: From the Centers for Disease Control and Prevention, National Center for Health Statistics, National Vital Statistics System*.

[27] Wyns C., De Geyter C., Calhaz-Jorge C. (2022). ART in Europe, 2018: Results Generated From European Registries by ESHRE. *Human Reproduction Open*.

[28] Rani G., Goswami S., Chattopadhyay R., Ghosh S., Chakravarty B., Ganesh A. (2015). Live Birth in a 50 Year-Old Woman Following In Vitro Fertilization-Embryo Transfer With Autologous Oocytes: A Rare Case Report. *Fertility and Sterility*.

[29] Wu T. W., Tsai H. D., Huang H. C. (2022). Rare Live Birth to a 48 Year-Old Woman After Embryo Transfer With Autologous Oocyte: A Case Report. *Taiwanese Journal of Obstetrics & Gynecology*.

[30] Nicopoullos J. D., Wren M., Abdalla H. (2015). Treatment and Preservation at the Extremes of Reproductive Age: A Case Report Outlining the Ethical Dilemmas. *Journal of Assisted Reproduction and Genetics*.

[31] Ubaldi F. M., Cimadomo D., Vaiarelli A. (2019). Advanced Maternal Age in IVF: Still a Challenge? The Present and the Future of Its Treatment. *Frontiers in Endocrinology*.

[32] Poli M., Capalbo A. (2021). Oocyte Cryopreservation at a Young Age Provides an Effective Strategy for Expanding Fertile Lifespan. *Frontiers in Reproductive Health*.

[33] Sabbagh R., Meyers A., Korkidakis A. (2025). Pregnancy Outcomes With Increasing Maternal Age, Greater Than 40 Years, in Donor Oocyte Cycles. *Human Reproduction*.

